# End-to-End Monocular Range Estimation for Forward Collision Warning

**DOI:** 10.3390/s20205941

**Published:** 2020-10-21

**Authors:** Jie Tang, Jian Li

**Affiliations:** College of Intelligence Science, National University of Defense Technology, Changsha 410073, China; kakaxi314@nudt.edu.cn

**Keywords:** end-to-end learning, range estimation, forward collision warning, convolutional neural networks

## Abstract

Estimating range to the closest object in front is the core component of the forward collision warning (FCW) system. Previous monocular range estimation methods mostly involve two sequential steps of object detection and range estimation. As a result, they are only effective for objects from specific categories relying on expensive object-level annotation for training, but not for unseen categories. In this paper, we present an end-to-end deep learning architecture to solve the above problems. Specifically, we represent the target range as a weighted sum of a set of potential distances. These potential distances are generated by inverse perspective projection based on intrinsic and extrinsic camera parameters, while a deep neural network predicts the corresponding weights of these distances. The whole architecture is optimized towards the range estimation task directly in an end-to-end manner with only the target range as supervision. As object category is not restricted in the training stage, the proposed method can generalize to objects with unseen categories. Furthermore, camera parameters are explicitly considered in the proposed method, making it able to generalize to images taken with different cameras and novel views. Additionally, the proposed method is not a pure black box, but provides partial interpretability by visualizing the produced weights to see which part of the image dominates the final result. We conduct experiments to verify the above properties of the proposed method on synthetic and real-world collected data.

## 1. Introduction

Range estimation, estimating distance from ego to the closest object in front, is the core component of the forward collision warning (FCW) system. Based on the estimated range and time to collision which can be computed by ranges in multi-frame, FCW can be made for automobile robot and advanced driver assistant systems (ADAS) to avoid potential collisions [1,2,3]. Although radar or LiDAR sensors can provide accurate range measurements, they are too heavy and expensive for small robots and ADAS. Estimating range from a single monocular image is a convenient and cheap solution, which is favored and widely adopted by commercial products, such as the famous Mobileye’s ADAS (https://www.mobileye.com/our-technology/adas/). In addition to the usage in industrial applications, predicting range from a monocular image is a challenging ill-posed problem with great value for academic research.

Due to the ambiguity of distance estimation from a monocular image, especially on the global scale [4], geometry relationship of perspective projection is often used to facilitate this estimation. Two cues are mostly considered in traditional approaches. One is the known vehicle size, in which the object category is constrained as a vehicle with a fixed size. According to the size of the detected vehicle in the image, the target range can be inferred through perspective projection [5,6,7]. However, considering different subcategories of vehicles like car, SUV, bus, and truck, the size of a vehicle has a large variance that could lead to a large error in the estimated range. The other cue is the planar road surface assumption. Typically, an object is detected in the image and the original image is converted to an Inverse Perspective Mapping (IPM) image to achieve distance along the forward direction [8,9,10,11]. Compared with the known vehicle size, planar road surface assumption is more general and presents more accurate results [12]. In general, all these traditional approaches need to detect the object first and then estimate the distance to this object by utilizing perspective projection with the chosen cue.

Recently, deep learning methods have achieved great success in the computer vision community. For range estimation, prior works [13,14,15] have proposed object-specific end-to-end deep learning frameworks. They follow the multi-task learning scheme to simultaneously detect objects and estimate the corresponding range for each object by direct regression. Compared to separately hand-craft designing or optimizing each step in traditional approaches, end-to-end manner of deep learning methods can train all components jointly with the learned features adapting to the task of interest and achieve superior performance. However, like traditional approaches, they can only estimate ranges for objects from some limited given categories, which still does not fulfill the requirement of FCW to respond to various objects (including unseen categories in the training stage).

In contrast to previous methods of mostly estimating ranges to all the specific objects in the image, our method is class-agnostic and only yields range to the closest object in the preset collision region, which is the real requirement of FCW. We express the target range as a weighted sum of a set of potential distances. These distances are from a distance map generated by inverse perspective projection based on intrinsic and extrinsic camera parameters, while the corresponding weights are from the weight map produced by a deep neural network. Unlike prior work [13] where the range is directly regressed with camera information ignored when deploying, camera parameters are explicitly embedded in our distance map generation. As demonstrated by [16,17,18] that explicitly implementing camera parameters into network can improve generalization capability, while conventional convolutional neural network (CNN) based direct regression methods tend to overfit dataset that can not generalize to new cameras and novel views. At the same time, the whole architecture of our method is differentiable, which also enables the merits of end-to-end learning.

Towards this end, we propose a novel network structure to predict the corresponding weights of the generated distances for range estimation. Specifically, following the planar road surface assumption, we first utilize inverse perspective projection to generate a distance map, in which each pixel representing the distance to a point on road surface. Then, the weight map is generated by a U-Net structure with the same resolution as the distance map. Considering that the convolutional layer can only provide spatially-agnostic operation in local, it is difficult for a CNN structure to learn the point that what we want is the range to the closest object, when multiple objects are in front. Therefore, we use fully convolutional networks as the encoder and decoder parts of the U-Net structure and employ fully connected layers on the encoded features to provide spatially-specific operations and global receptive field. Finally, we mask both the distance map and weight map to just remain pixels in the preset collision region to estimate range as a weighted sum of distances. The whole network is trained in an end-to-end manner with only the target range as supervision, which avoids expensive object-level annotation such as class label and bounding box and encourages the network to be class-agnostic as well.

We process a synthetic dataset to build a dataset with accurate ground truth range for training and testing. In addition to synthetic data, we also collect data in the real world by our autonomous driving vehicle to verify our method. Our method can generalize to objects of unseen categories, different cameras, and novel views. It also presents superior performance when compared with an IPM-based two-step method. Moreover, our method is not a pure black box but provides partial interpretability because the produced weight map can easily be visualized to indicate which part of the image dominates the estimated range.

## 2. Approach

### 2.1. Overview

**Figure 1:**
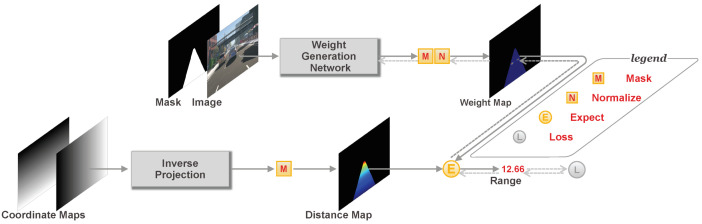
Overview of architecture. The range is represented as a weighted sum of a set of potential distances and the whole architecture consists of weight map generation and distance map generation. The solid lines with arrows represent the forward implementation of our method, while the dashed lines with arrows indicate the loss calculation and back-propagation in the training stage.

Our method is an end-to-end deep learning architecture that directly estimates range to the closest object in front for forward collision warning (FCW). The pipeline of the proposed method is illustrated in Figure 1. Based on the planar road surface assumption, we use inverse perspective projection to convert the U,V coordinate maps of an image to a distance map, in which each pixel value corresponds to the distance to the road surface along the forward direction. The range to be estimated is formalized as a weighted sum of these distances. The corresponding weights are generated from a weight generation network with a single image and the mask of the collision region as input. The whole network is differentiable and can be supervised only by the ground truth range.

### 2.2. Distance Map Generation

Estimating distance from a single monocular image is severely under-constrained. The estimated distance would have inherent ambiguity, especially on the global scale. Some assumptions are needed to infer distance directly from image coordinates. Our method follows the planar road surface assumption to generate a distance map through inverse perspective projection. The planar road surface assumption assumes that the road surface is a plane and objects are on the road, which is general and has no restriction on the category of the object.

**Figure 2:**
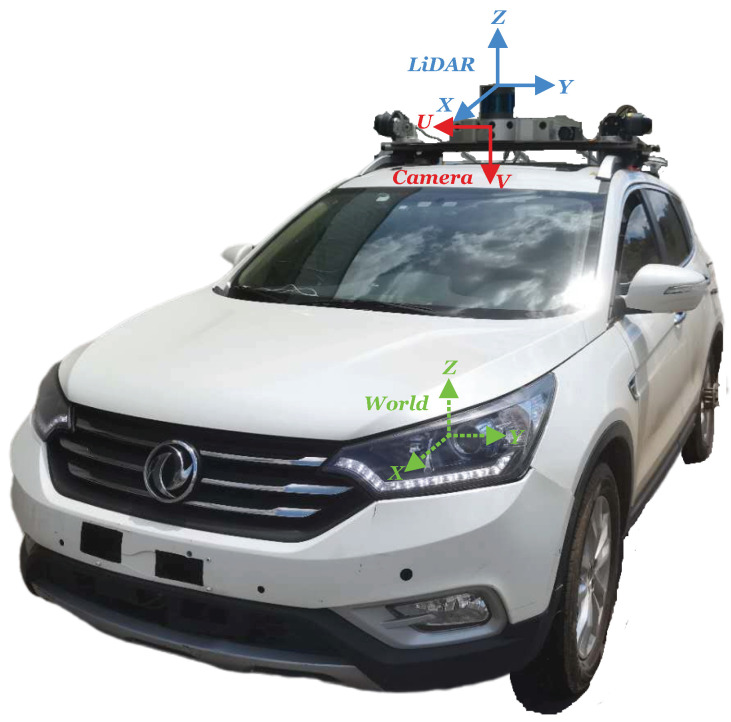
Our autonomous driving vehicle with image coordinate system, LiDAR coordinate system, and world coordinate system in our setting.

Under the pinhole camera model, the 3D scene is mapped onto a 2D image plane. Thus, each pixel ${\left(u,v\right)}^{T}$ in an image corresponds to a point ${\left(x,y,z\right)}^{T}$ in 3D space. The camera coordination system and the world coordinate system in our setting are illustrated in Figure 2. The center point of the world coordinate system is on the road plane and straightly beneath the camera with *Z* axis perpendicular to the road surface. The correspondence relationship between these two coordinate systems can be expressed by perspective projection:

(1) $$s\cdot {\left(u,v,1\right)}^{T}=K\cdot \left[R|T\right]\cdot {\left(x,y,z,1\right)}^{T},$$

where *K* and $\left[R|T\right]$ are intrinsic and extrinsic camera parameters respectively and *s* is the scalar projective parameter. With the planar road surface assumption, for all the 3D points on the road surface, coordinates *z* in the world coordinate system equal to 0, which can be removed from Equation (1). Then,

(2) $$s\cdot {\left(u,v,1\right)}^{T}=P\cdot {\left(x,y,1\right)}^{T}.$$

Here, *P* is a 3×3 projection matrix. For each pixel ${\left(u,v\right)}^{T}$ in the image coordinate, we use inverse projection to calculate the corresponding ${\left(x,y\right)}^{T}$, which indicates a point on the road surface with *x* representing the distance along the forward direction.

Some traditional methods [8,9,10] use perspective projection to convert the original image to an image in bird’s eye view which is usually called an Inverse Perspective Mapping (IPM) image. Then the distance information is obtained from this interpolated IPM image. In contrast, the proposed method works on the original image plane to generate a distance map with the same resolution as the original image. One example of the generated distance map is visualized in Figure 1. The value on each pixel of distance map is the calculated *x* from the inverse perspective projection, representing the distance to the road surface. As we are only concerned with the object in front of the ego vehicle, which may cause a forward collision, we set a potential collision region to mask the distance map by limiting the *y* coordinate according to the width of the ego vehicle and the *x* coordinate according to the farthest distance we care about. Finally, the masked distance map consists of a set of potential distances for the target range. More details of the mask generation are introduced in Section 3.

For this distance map generation, even though the required intrinsic and extrinsic camera parameters can be offline calibrated, the vehicle motion may cause perturbation on the extrinsic parameters. The extrinsic parameters can also be estimated on-the-fly. For example, in [19], the extrinsic parameters are estimated based on the parallel lane detection. How to obtain accurate extrinsic parameters for inverse projection is out of the scope of this paper. In our method, we assume we have both the accurate intrinsic and extrinsic camera parameters.

### 2.3. Weight Map Generation

**Figure 3:**
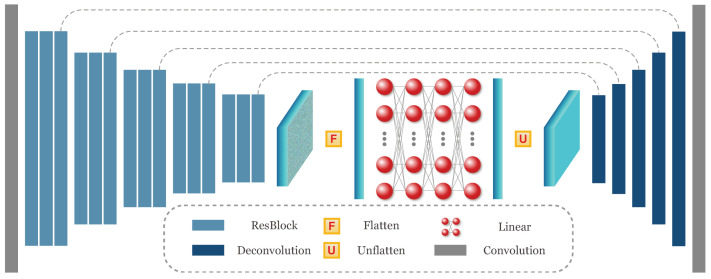
The network structure of weight generation network. The U-Net structure is an encoder-decoder network. The encoder and decoder parts consist of fully convolutional networks. Fully connected layers are applied to the flattened encoded features to provide spatial position information. See the text for more details.

We consider the target range as a weighted sum of a set of distances within the masked region. Thus, the range is obtained by dot product of the distance map and a weight map. The weight map is generated by a deep neural network with the same resolution as the distance map. In principle, any off-the-shelf dense prediction architecture can be adopted for such weight map generation. In our method, as shown in Figure 3, we employ a novel U-Net structure network to generate the weight map by extracting features on multi-resolution.

Typically, the U-Net structure network [20] is an encoder-decoder network. We adopt fully convolutional structures for the encoder and decoder parts. For the encoder part, we firstly apply a 5 × 5 convolution layer on the input images. Then, each time a convolution layer with the stride of 2 is adopted to reduce the resolution of the feature map. For each resolution, 3 stacked ResBlocks [21] are used to extract features. The ResBlock consists of two sequential 3×3 convolution layers with a skip layer. The resolution of the encoded feature is 1/32 of the original resolution. For the decoder part, we use a sequence of deconvolution layers with the stride of 2 to increase the resolution of the feature map. Finally, a 1 × 1 convolution layer is used to map the feature map to single-channel output. In this U-Net structure, for each resolution, we employ an additional skip connection to fuse the feature maps of the same resolution from the encoder to the decoder. Each convolution or deconvolution layer is followed by a batch normalization [22] and a ReLU [23] layer. As shown in Figure 1, we concatenate the color image and a mask of preset collision region as the input of the U-Net structure. The mask added here is to force the network to pay attention to the region of interest.

As it is possible to have multiple objects in front, the network needs to learn the point that the desired range is the one to the closest object. However, this point is difficult to learn by a fully convolutional network [24], since the parameter sharing mechanism of convolution layer can only provide spatially-agnostic operation with local receptive field. To make the network aware of the spatial position information, we apply three fully connected layers which can assign unique parameters for each spatial position on the encoded feature. For the encoded feature map, we keep the channel dimension unchanged and only flatten the spatial dimension, i.e., the height and width dimension. Then the linear operation of the fully connected layer works on the flattened feature and is shared among channels. By sharing parameters among channels, these fully connected layers can introduce spatial position information without causing too many parameters. In addition to spatial position information, these fully connected layers also provide global perspective field for the network. We apply a dropout [25], layer normalization [26], and ReLU [23] layers after each fully connected layer. The produced features from these fully connected layers are reshaped back (unflattened) to feature map with 2D spatial dimensions and fed to the decoder part of the network.

In our method, these generated weights work as probabilities of corresponding distances for the expected range. Thus, these weights should be non-negative and their sum should be equal to 1. We adopt a softplus [27] layer on the single-channel output of the U-Net structure to yield a weight map with all the values positive. The weight map is then masked by the preset collision region. Finally, we normalize the masked weights to make them suitable as probabilities.

### 2.4. End-to-End Learning

Our method learns the range from a single image in an end-to-end manner. This end-to-end learning is optimized by minimizing the difference between estimation and ground truth range. Unlike previous end-to-end object-specific distance estimation methods [13,14,15] that require expensive object-level annotations as supervision, our method is only trained with ground truth range and has no restriction on object category, which makes it possible to generalize to objects of unseen categories. Meanwhile, we have no direct supervision on the predicted weight map. The weight map is generated by the weight generation network which is only supervised to produce accurate range estimation. Since the weight map is a single channel image, as shown in Figure 1, it can easily be visualized to see which part of the image dominates the estimated range. Thus, our method is not a pure black box but has partial interpretability.

### 2.5. Training Settings

We adopt mean absolute error (MAE) as the training loss to train our range estimation network. We process a synthetic dataset to build a dataset with accurate ground truth range for training. In total, about 170k samples are used for training. More details about the dataset preparation are introduced in Section 3.1. Color image with bottom-center cropped 320×960 resolution and the corresponding mask of the preset collision region are used as the input of our network. The code is implemented in Pytorch and trained from scratch in a GPU server with 8 Nvidia GTX 2080Ti GPUs. The batch size on each GPU is 8 and the batch norm layers in our network are synchronized across GPUs. We utilize ADAM [28] as the optimizer with an original learning rate of $10^{-3}$ and weight decay of $10^{-6}$. The network is trained for 80 epochs while the learning rate drops to half at the 40th and 60th epochs.

## 3. Experiment Setup

One of the properties of data-driven methods especially deep learning methods is data-hungry. A large dataset is usually necessary for training. However, it is difficult to obtain accurate range information in the real world, especially considering that it must satisfy the planar road surface assumption adopted in our method. To have a large enough dataset with little noise and error on ground truth range, we process a synthetic dataset to build a dataset for training and testing. We also collect data in the real world by our autonomous driving vehicle with ground truth range from LiDAR to verify our method.

### 3.1. Synthetic Dataset

The synthetic Apollo dataset (https://apollo.auto/synthetic.html) is one of the largest photo-realistic synthetic datasets for autonomous driving currently. The dataset is created by Unity 3D engine with various distinct virtual scenes, including highways, urban, residential, downtown, and indoor parking garage. The dataset also contains plenty of environmental variations, such as different times of day, different weather conditions, different objects, and varied road surface qualities. In addition to the synthesized color image, it provides extensive ground truth data, such as 2D/3D object data, semantic/instance-level segmentation, depth, and 3D lane line data. We choose data in good light conditions (data with time at 9:00, 13:00, and 14:00) and good road surface quality (referred to as ‘No Degradation’ in this dataset) for our experiment.

Camera parameters are required by our method. The intrinsic camera parameters are provided by the dataset. We calculate the extrinsic parameters, which transfer the coordinate from the world coordinate system to the camera coordinate system shown in Figure 2. In particular, we first find the depths of all the pixels whose semantic labels are ‘road’ from the depth image and semantic segmentation image. Based on camera projection, we convert these pixels with depth to a set of 3D points. Then we use RANSAC to fit a plane from these 3D points. Based on the normal of the road plane, we calculate the pitch and roll angles and the height to the road plane of the ego camera. Finally, we use these angels and height to build the $\left[R|T\right]$ matrix as the extrinsic camera parameters. In this process, we remove the image from the dataset if we find these 3D points have a sufficient number of outliers of the fitted plane, which indicated that the sample violates the planar road surface assumption.

The target range to the closest object depends on the preset collision region. The collision region is a rectangle space on the road, whose width is the width of the ego vehicle, and height is the farthest distance concerned. The rectangle is located in front of the ego vehicle, and the position may have an offset angle to the forward direction of the camera due to the yaw angle between the camera and vehicle. We project the collision region into image plane to get the potential collision mask. Thus, the collision region is decided by the width of the ego vehicle, the farthest distance concerned, and the offset angle to the forward direction of camera. For this synthetic Apollo dataset, we randomly adjust these three parameters (the vehicle width from 1.5 m to 2.5 m, the farthest distance from 80 m to 90 m, the offset angle from $-10^{\circ }$ to $+10^{\circ }$) to provide different collision regions. In this way, a single image may have different target ranges corresponding to different collision regions. It can be seen as data augmentation and forces our method to be robust to different masks.

**Figure 4:**
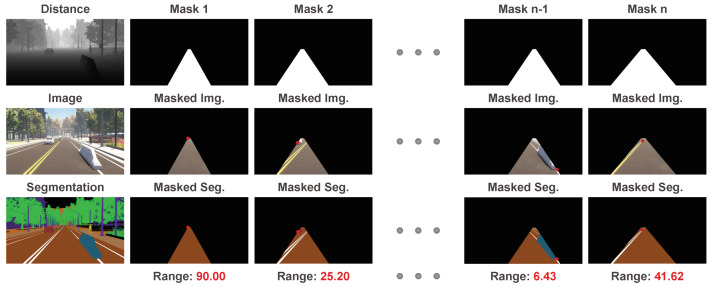
Processed Apollo dataset. These masks represent different preset collision regions. Even for the same color image, different collision regions may induce different target ranges.

In the collision region, we take the closest distance among the pixels whose semantic labels are not ‘road’ as the target range. As there are confusing situations in the synthetic Apollo dataset, e.g., leaves on the road, which may not cause a forward collision, we remove the sample from the dataset if the semantic label of this closest-distance pixel is ‘vegetation’. We also remove the sample from the dataset if the semantic label of this closest-distance pixel is ‘terrian’, which may be just a small bump on the road. In Figure 4, we present some examples from the generated dataset. The closest-distance pixel is marked with a red dot. In total, there are 190k samples with color images, camera parameters, collision regions, and ground truth ranges. We randomly select 90% samples as the train set and the remaining as test set.

### 3.2. Real-World Data Collection

We also collect data in the real world by our autonomous driving vehicle to verify our method. Our autonomous driving vehicle has one 12 mm lens camera and one 128-line LiDAR mounted as shown in Figure 2. The camera and LiDAR are synchronized with a trigger signal to guarantee LiDAR measures range in the forward direction when the image is captured by the camera. In order to obtain measurements as accurately as possible, our ego vehicle is parked on a flat road to collect data and avoid the effect of ego-motion. In the process of data collection, the scene in front is almost fixed or dynamic objects move at a very low speed. The intrinsic and extrinsic camera parameters (camera coordinate system to LiDAR coordinate system and camera coordinate system to world coordinate system) were calibrated before data collection. The collision region for the data collected by our autonomous driving vehicle is determined by the vehicle width of 1.8 m, the farthest distance of 85 m, and the offset angle of $0^{\circ }$.

## 4. Results and Analyses

Our method produces range to the closest object in the front preset collision region for FCW. We demonstrate the properties of our method and compare its performance with a traditional IPM-based method.

### 4.1. Interpretability

Our method is not a pure black box but has partial interpretability. The estimated range is produced as a weighted sum by a distance map and weight map. The weight map, which is generated by a U-Net structure, is a single-channel image of the same resolution with the input color image. It works as a probability map to yield the expected range. By visualizing the generated weight map, we can take a look at which part of the image the weight map focused on to produce the estimated range.

**Figure 5:**
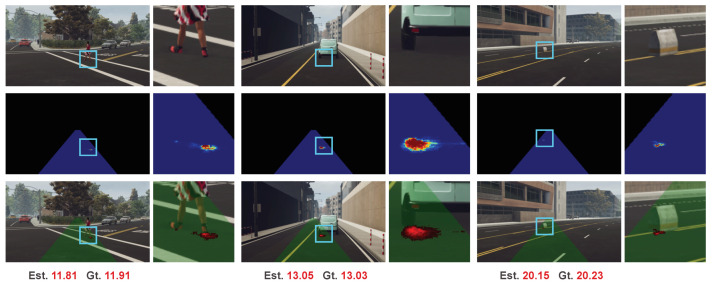
Visualization of generated weight maps. The first row is the color image. The second row is the colorized weighted map. The last row is the color image superimposed on the preset collision region and the colorized weighted map. This interpretable representation of the last row will be used to illustrate experimental results in the following. We use cyan rectangles to highlight the notable regions and zoom in these regions for better visualization.

We illustrate several examples in Figure 5. It is interesting that even no direct object-level supervision such as bounding box is used, our network successfully learns to give a high response on the intersection region of the target object and road surface. Compared to the other part in the preset collision region (the green region in the last row), the distances in the intersection region of the distance map are closer to the target range. Thus, it is reasonable to give larger weights on this intersection region for range estimation. From this view, our method shares the same philosophy with traditional IPM-based methods that the intersection region of the target object and road surface is found for range estimation. However, in contrast to achieving the intersection region by object detection in traditional IPM-based methods, our method implicitly learns to give larger weights on this intersection region in an end-to-end manner towards the true range estimation task.

### 4.2. Class-Agnostic Property

**Figure 6:**
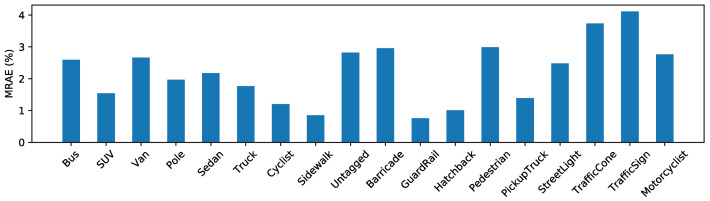
Performance on target objects of various classes.

Previous monocular range estimation methods mostly involve two sequential steps of object detection and range estimation that can only estimate ranges for objects from some limited given categories. Our method is only supervised by the target range and has no restriction on the object category. This encourages our method to be class-agnostic, which can work on objects of various categories, even unseen categories in the training stage. In Figure 6, we illustrate the mean relative absolute error (MRAE) on the test set. We can see that our method performs well on objects from various classes.

**Figure 7:**
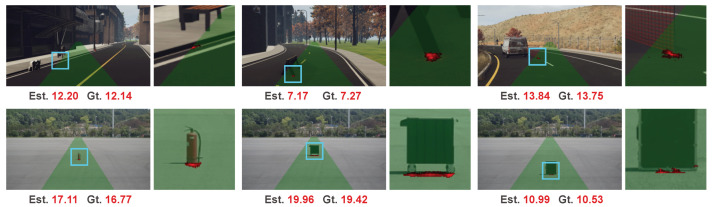
Results on objects of various categories.

We illustrate some results in Figure 7. The top row is the results of images from the test set of the processed synthetic Apollo dataset. Our method works well on these examples which are more flexible and rarer than typical objects such as cars and pedestrians. The bottom row shows the results on some real-world collected data. These objects, including fire-extinguisher, cart, and suitcase, are categories that rarely appear in traffic scenes and are not included in the train set. We can see our method can still work on these unseen categories, which demonstrates the class-agnostic property of our method.

### 4.3. Generalization Capability

**Figure 8:**
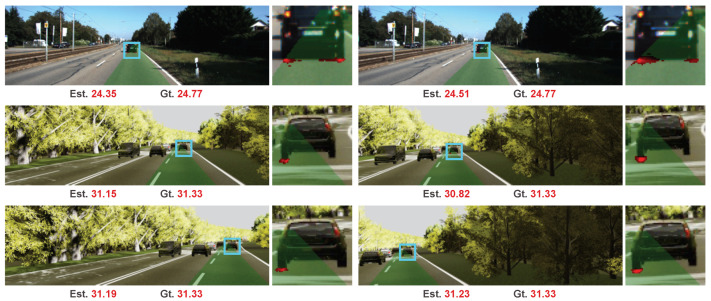
Generalization capability. These six images are from KITTI and virtual KITTI datasets. They are collected with different cameras and views than the training dataset.

Our method is trained on the processed synthetic Apollo dataset. As the intrinsic and extrinsic camera parameters have been explicitly embedded in the distance map generation of our method, our method can generalize to images captured from different cameras and novel views, while methods estimating range by hard regression with camera parameters ignored tends to overfit dataset and can not generalize. We selected some examples from the KITTI dataset [29] and virtual KITTI dataset [30], and show the results in Figure 8. The first row is the paired images from KITTI dataset [29] which are captured in the traffic scene by a stereo camera mounted on an autonomous driving vehicle. The following two rows are the images from virtual KITTI dataset [30], which is a synthetic dataset. The second row is two images captured by cameras mounted with left 15-degree and right 15-degree offsets to the forward direction. The two images in the last row are from cameras with left 30-degree and right 30-degree offsets. We can see our method works well on these six images, even though they are captured with different cameras and different views than images in the train set.

### 4.4. Closest Object

The goal of our method is to estimate the range to the closest object for FCW. When there are multiple objects in the potential collision region, we expect our method to provide the range to the closest object, without being disturbed by other objects that are farther away. In order to encourage our method to learn this point, we apply fully connected layers on the encoded features to provide spatially-specific operations and global receptive field.

**Figure 9:**
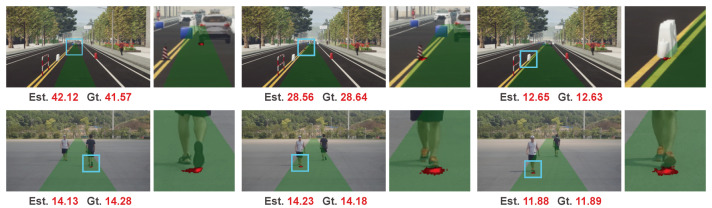
Results on images with multiple objects in front.

We test our method on some examples with multiple objects in front and show results in Figure 9. The top row is the results of one image with three different collision regions. The different collision regions contain different numbers of objects with different target ranges. We also collect data with two pedestrians walking in front shown in the bottom row. For each image, we can see from the superimposed weight maps that our method can focus on the closest object to estimate range.

### 4.5. Comparison

**Figure 10:**
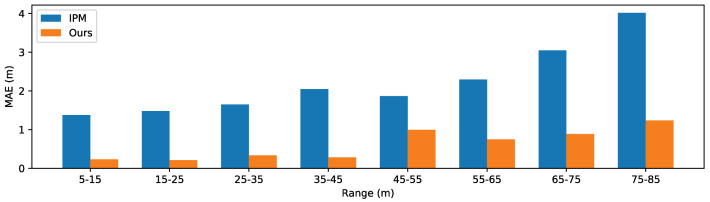
Performance on cars in the test set of the processed synthetic Apollo dataset.

As analyzed in Section 4.1, by visualizing the generated weight map, we find our method shares the same philosophy with the traditional IPM-based method. Thus, we compare the performance of our method and traditional IPM-based method on the test set of the processed synthetic Apollo dataset. For the IPM-based method, we first use the released code and model of [31] to detect the car in front which is one of the top-ranked 2D car detection methods in KITTI object detection benchmark (http://www.cvlibs.net/datasets/kitti/eval_object.php?obj_benchmark=2d). With the same camera parameters as our method, we then project the center point of the bottom side of the bounding box into bird’s eye view coordinate and interpolate distance from the IPM image. Since the object category is constrained as car for the object detection model of [31], we only consider the samples in the test set whose closest object in the preset collision region belongs to the classes of ‘bus’, ‘SUV’, ‘van’, ‘sedan’, ‘truck’, ‘hatchback’, and ‘pickuptruck’. We ignore the sample if the closest car is not successfully detected by [31]. We show the MAE of these two methods in Figure 10. Compared with the IPM-based method, our method has a lower MAE over all the ranges.

**Figure 11:**
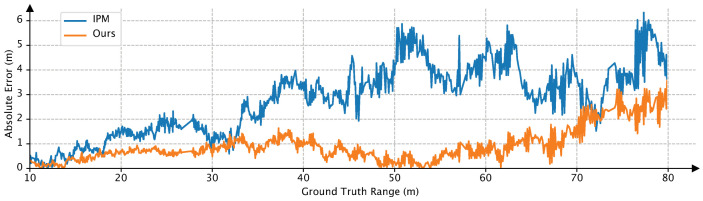
Performance on a data sequence collected by our autonomous driving vehicle.

We also collect a data sequence by our autonomous driving vehicle with a car moving in front to evaluate the performance of our method and traditional IPM-based method. Figure 11 depicts the quantitative results. The horizontal axis is the ground truth range obtained from the LiDAR sensor and the vertical axis is the absolute error between the estimate and ground truth range. We can see that compared with the IPM-based method, our method has a better performance, especially when the target car is far away.

**Figure 12:**
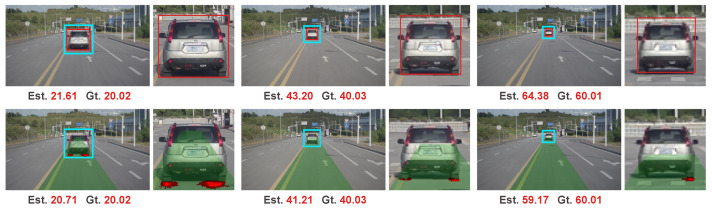
Samples of results in our collected sequence. The results of the Inverse Perspective Mapping (IPM)-based method are shown in the top row. The results of our method are shown in the bottom row.

We illustrate some samples of the results of our collected data in Figure 12. The top row is the results of the IPM-based method and the bottom row is the results of our method. The bounding boxes are drawn with red rectangles for the IPM-based method. We can see that even the target car is successfully detected in the IPM-based method, its performance is still worse than our method with a larger error on the estimated range. We attribute the superior performance of our method to the end-to-end learning. Compared to end-to-end learning whose optimization objective is towards the true task, the IPM based method is a two-step method with the first step optimized to accurate bounding box for object detection which is a proxy task. Object detection tends to produce relatively accurate bounding boxes, for example, satisfying the criteria of IoU>0.7 in KITTI object detection benchmark. However, it is still difficult to achieve the precise intersection region of the object and road surface from the detected bounding box.

### 4.6. Failure Cases

**Figure 13:**
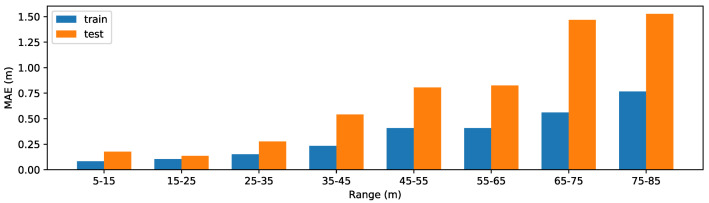
Mean absolute error (MAE) (in m) on the train set and test set under different ranges.

Our method is trained on the train set of the processed synthetic Apollo dataset. In Figure 13, we illustrate the mean absolute error (MAE) on the train set and the test set under different ranges. We can see that distant objects tend to have larger errors.

**Figure 14:**
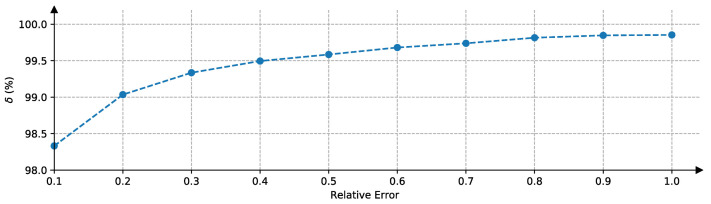
δ corresponding to the threshold of relative error in the test set.

As MAE only demonstrates the mean performance of our method on the dataset, we also plot the δ of our method on the test set in Figure 14. δ means the percentage of samples where the relative error is less a threshold. We can see that for more than 98% of samples, the relative error is less than 0.1. While even the threshold of relative error goes up to 1.0, δ is still less than 100%. This suggests that there are cases in which our method completely fails.

**Figure 15:**
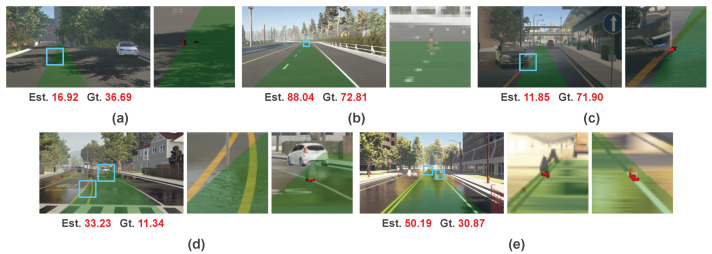
Examples of five types of failure cases of our method. See the text for more details.

Thanks to the partial interpretability of our method, we can classify and analyze these failure cases. In general, the failure cases of our method can be classified as five types. We illustrate these five types of failure cases in Figure 15. The first type is complex road surface, which may be caused by marks or shadow of trees. As shown in Figure 15a, our method is affected by the shadow on the road surface and treats it as the target object. The second type is small distant object shown in Figure 15b. The object only occupies a few pixels and is missed by our method. The third type is that the object is almost at the boundary of the preset collision region as shown in Figure 15c. In this ambiguous position, our method is confused whether to respond to this object. The fourth type is that our method does not respond to the closest object, but a farther object. As shown in Figure 15d, it mostly occurs when the perception of the closest object is difficult, such as a very thin pole. The fifth type is that our method responds to multiple objects as shown in Figure 15e. In our method, we employ a U-Net structure network to produce the weight map. Even though we apply fully connected layers on the encoded features to provide spatial position information, the proposed method still does not have strict constraints to ensure that the generated weight map only responds to the single closest object.

## 5. Conclusions and Future Work

In this paper, we propose a novel end-to-end network to estimate the range to the closest object in front from a monocular image for forward collision warning (FCW). The target range is represented as a weighted sum of a set of potential distances. These distances are generated by inverse perspective projection under the planar road surface assumption and the corresponding weights are produced by a deep neural network. The proposed method is end-to-end trained with only the ground truth range as supervision. Experimental results on synthetic data and real world collected data show that the proposed method has several properties favored by FCW: (1) Class-agnostic property for objects of various categories; (2) generalization capability for images from new cameras and novel views; (3) partial interpretability indicating which part of the image dominates the result; and (4) superior performance to the IPM-based method.

In the future, we plan to collect a large dataset in the real world with accurate ground truth range to fine-tune our model, as the model trained by a synthetic dataset could still easily fail in a real complex scene due to the gap between these two domains. Furthermore, we assume the planar road surface and accurate extrinsic camera parameters in our method. Fitting for non-planar road and estimating extrinsic camera parameters on-the-fly even as a trainable component of the method would be interesting and meaningful extensions of this work.
